# Correction: Genomic regions of current low hybridisation mark long-term barriers to gene flow in scarce swallowtail butterflies

**DOI:** 10.1371/journal.pgen.1012000

**Published:** 2026-01-16

**Authors:** The PLOS Genetics Staff

## Notice of Republication

This article was republished on December 10, 2025, to correct an error in XML. The colours of the figures were displaying incorrectly. The publisher apologizes for the errors.
