# Supplementary material for: Genomic regions of current low hybridisation mark long-term barriers to gene flow in scarce swallowtail butterflies
Source: PLoS Genet. 2025 Apr 10;21(4):e1011655. doi: 10.1371/journal.pgen.1011655 (PMC12040345; doi:10.1371/journal.pgen.1011655)
Supplement: S1 Appendix — (PDF) [file pgen.1011655.s001.pdf]

APPENDIX

In the Appendix of Baird [1, Equation A10], Barton expressed the per migrant contribution to the distribution of continuous tract length x for two infinite demes of coupling θ genomes, T rescaled generations after starting to exchange migrants, in terms of Kummer's confluent hypergeometric function ${}_1F_1\left(a;b;z\right)$ [2]:

$$F\left(x,T\right) = Te^{-x(1+\theta)T}{}_1F_1\left(\frac{\theta-1}{\theta+1};2;-T(1+\theta)(1-x)\right) \tag{A-1}$$

This can be re-arranged such that tract length only appears as an argument of the confluent hypergeometric function, not in the initial term, by using Kummer's first transformation:

$${}_1F_1\left(a;b;z\right) = e^{z}{}_1F_1\left(b-a;b;-z\right) \tag{A-2}$$

Applying transformation A-2 to the confluent hypergeometric function in A-1:

$${}_1F_1\left(\frac{\theta-1}{\theta+1};2;-T(1+\theta)(1-x)\right) = e^{-T(1+\theta)(1-x)}{}_1F_1\left(2-\frac{\theta-1}{\theta+1};2;T(1+\theta)(1-x)\right) \tag{A-3}$$

Multiplying both sides of A-3 by the initial term in A-1, and simplifying, tract length appears only in the confluent hypergeometric function:

$$F\left(x,T\right) = Te^{-T(1+\theta)}{}_1F_1\left(\frac{3+\theta}{1+\theta};2;T(1+\theta)(1-x)\right) \tag{A-4}$$

In this form of Barton's result the first argument of the confluent hypergeometric is the negative log-log gradient of the equilibrium distribution of tract length y from Barton [3, Eq (5)], clarifying the link between Barton's time T and equilibrium solutions. Integrating contributions A-1 since time of contact, the distribution of tract lengths per migrant found in either infinite deme is:

$$\int_0^T F(x,t)dt = (1+\theta)^{-2}x^{-\frac{3+\theta}{1+\theta}}; T \longrightarrow \infty \tag{A-5}$$

The left hand expression can be found by numerical integration for any time T, and becomes equal to the right hand equilibrium solution for progressively smaller tracts as time goes on.

References

1. Baird S. A simulation study of multilocus clines. Evolution. 1995;49(6):1038–1045.

2. Kummer, E. E. De integralibus quibusdam definitis et seriebus infinitis. Journal für Die Reine und Angewandte Mathematik. 1837;17:228-242.

3. Barton NH. Multilocus clines. Evolution. 1983; p. 454–471.
