# Supplementary material for: Genomic regions of current low hybridisation mark long-term barriers to gene flow in scarce swallowtail butterflies
Source: PLoS Genet. 2025 Apr 10;21(4):e1011655. doi: 10.1371/journal.pgen.1011655 (PMC12040345; doi:10.1371/journal.pgen.1011655)
Supplement: S1 Fig — (PDF) [file pgen.1011655.s003.pdf]

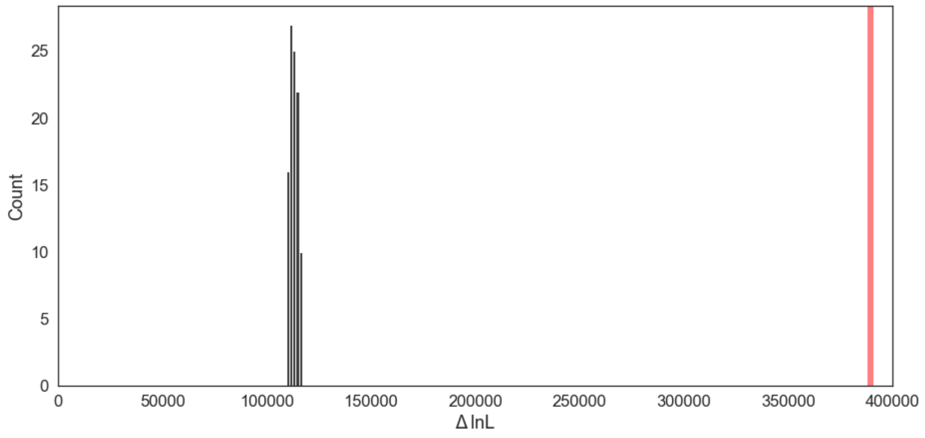

Figure S1 – The distribution of differences in log-likelihood ($\ln L$) between an isolation with migration (IM) model versus a model of strict divergence (DIV) fit to 100 replicates of data simulated under the best fitting empirical DIV model (black) and the empirical $\ln L$ (red). The improvement in fit of an IM model is much greater in the empirical data than in the simulations. See methods for simulation details.
