# Supplementary material for: Genomic regions of current low hybridisation mark long-term barriers to gene flow in scarce swallowtail butterflies
Source: PLoS Genet. 2025 Apr 10;21(4):e1011655. doi: 10.1371/journal.pgen.1011655 (PMC12040345; doi:10.1371/journal.pgen.1011655)
Supplement: S2 Fig — (PDF) [file pgen.1011655.s004.pdf]

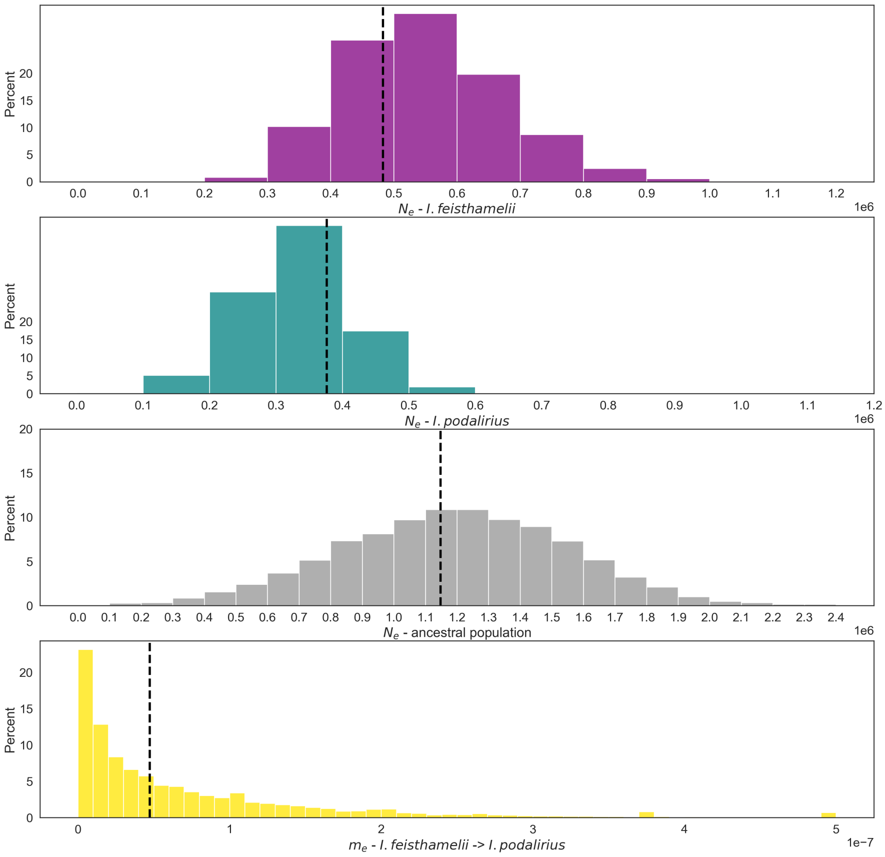

Figure S2 – The distributions of N_{fei} (purple), N_{pod} (teal), N_{anc} (grey), and $m_{e,i}$ (yellow) from *I. feisthamelii* into *I. podalirius* estimated in sliding windows using a 12x24x9x50 parameter grid. The vertical dashed lines indicate the global estimates of each parameter.
