# Supplementary material for: Genomic regions of current low hybridisation mark long-term barriers to gene flow in scarce swallowtail butterflies
Source: PLoS Genet. 2025 Apr 10;21(4):e1011655. doi: 10.1371/journal.pgen.1011655 (PMC12040345; doi:10.1371/journal.pgen.1011655)
Supplement: S3 Fig — (PDF) [file pgen.1011655.s005.pdf]

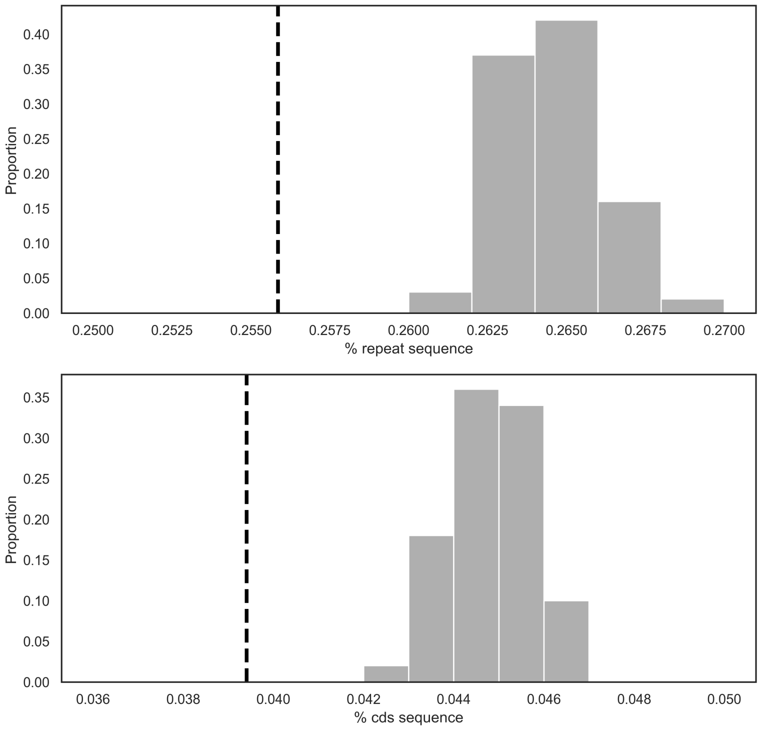

Figure S3 – The dashed lines indicate the mean density of repeats (top) and coding sequence (CDS) (bottom) in barrier regions defined via `gIMble`. Both are lower than the CDS and repeat proportion of data resampled at random using a circular resampling scheme.
