# Supplementary material for: Genomic regions of current low hybridisation mark long-term barriers to gene flow in scarce swallowtail butterflies
Source: PLoS Genet. 2025 Apr 10;21(4):e1011655. doi: 10.1371/journal.pgen.1011655 (PMC12040345; doi:10.1371/journal.pgen.1011655)
Supplement: S4 Fig — (PDF) [file pgen.1011655.s006.pdf]

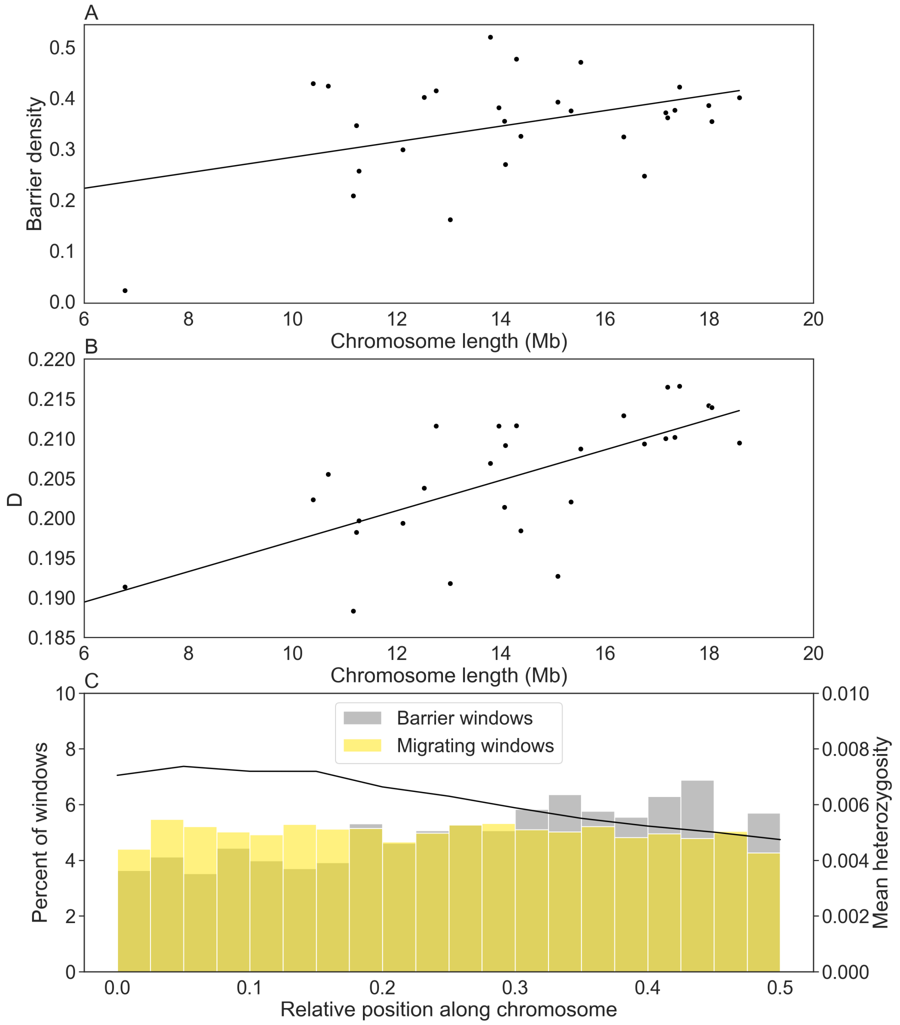

Figure S4 – A) Chromosome length (Mb) is positively correlated with barrier density (Pearson's $\rho = 0.425$, p = 0.0241). B) Chromosome length (Mb) is positively correlated with average D estimated within gIMble-defined windows (Pearson's $\rho = 0.74$, p = 4.84e-06). C) The distribution of relative positions from the telomeres (at 0) to chromosome centres (at 0.5) for `gIMble` barrier windows (grey) and non-barrier windows (yellow). The average heterozygosity for windows at different relative positions is shown by the black line. Barrier windows are further from the telomeres than non-barrier windows (circular bootstrap, p = 0.001).
