# Supplementary figures and images for: Genomic regions of current low hybridisation mark long-term barriers to gene flow in scarce swallowtail butterflies
Source: PLoS Genet. 2025 Apr 10;21(4):e1011655. doi: 10.1371/journal.pgen.1011655 (PMC12040345; doi:10.1371/journal.pgen.1011655)

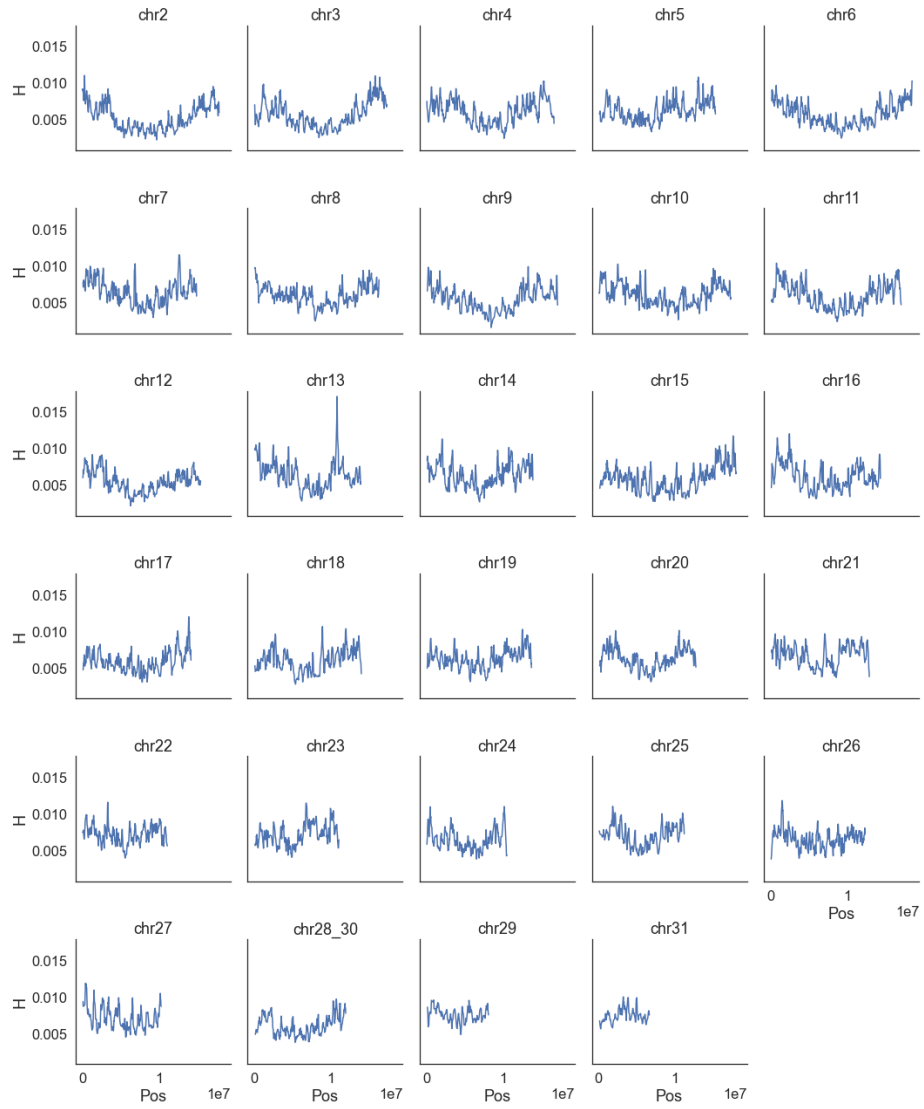

**Figure S5** – Mean heterozygosity (H) in sliding windows of minimum 50kb across all chromosomes.

Supplement: S5 Fig — (PDF) [file pgen.1011655.s007.pdf]

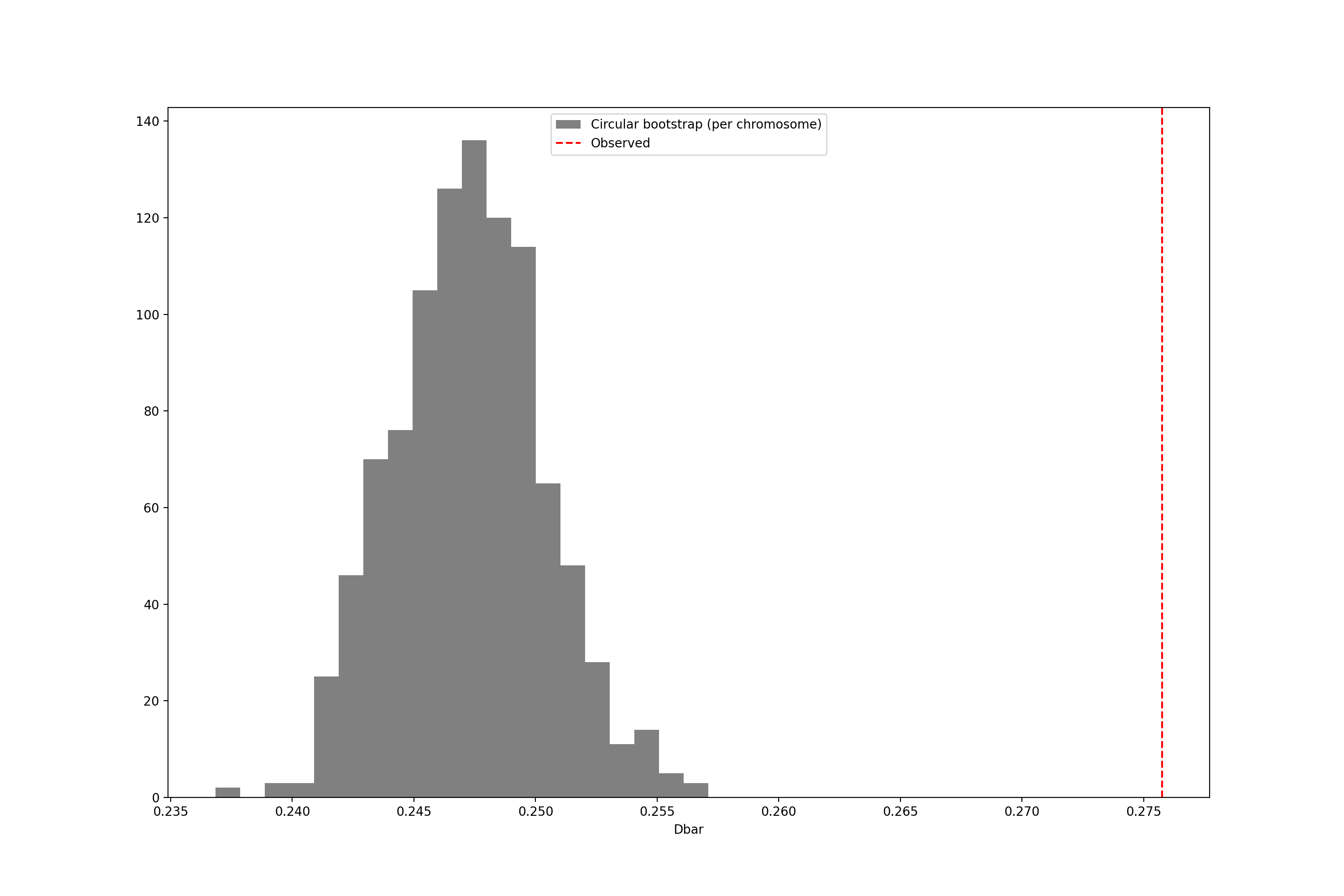

Supplement: S8 Fig — (PNG) [file pgen.1011655.s010.png]

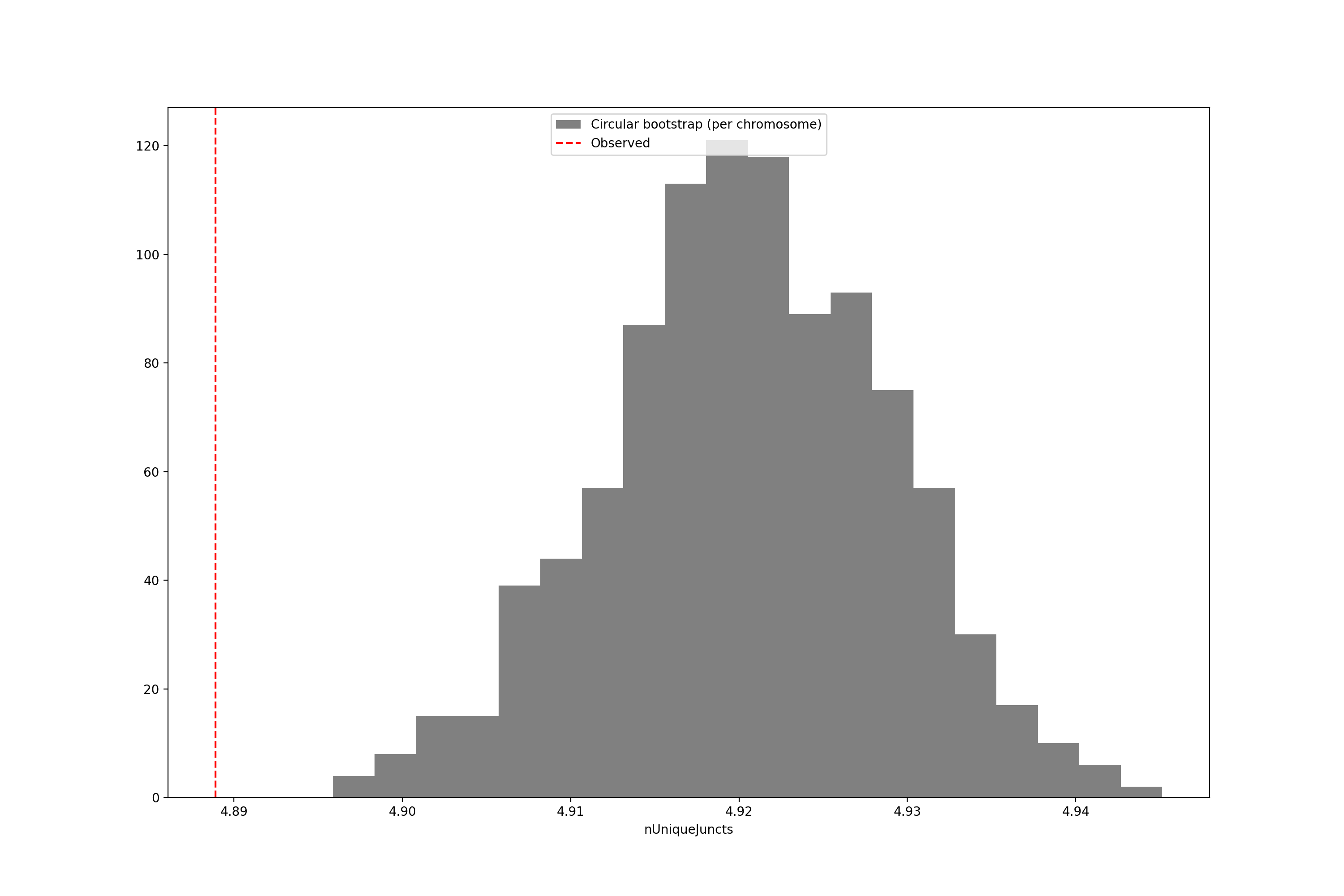

Supplement: S9 Fig — (PNG) [file pgen.1011655.s011.png]
