# Supplementary material for: Genomic regions of current low hybridisation mark long-term barriers to gene flow in scarce swallowtail butterflies
Source: PLoS Genet. 2025 Apr 10;21(4):e1011655. doi: 10.1371/journal.pgen.1011655 (PMC12040345; doi:10.1371/journal.pgen.1011655)
Supplement: S6 Fig — (PDF) [file pgen.1011655.s008.pdf]

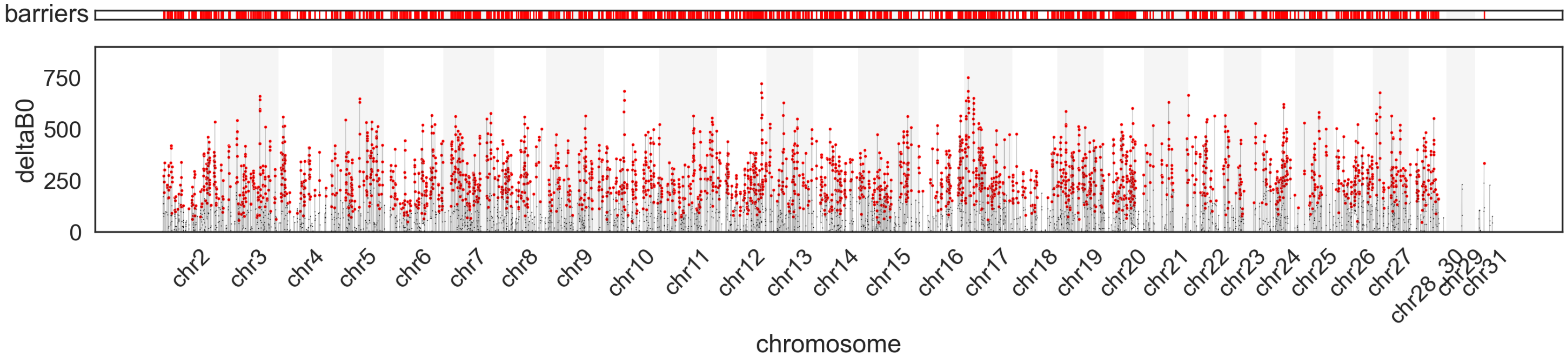

Figure S6 – Barriers to gene flow between *I. feisthamelii* and *I. podalirius.* The red bars in the top panel highlight the location of barrier windows. The bottom panel shows for each ***gIMble*** window, the difference in $\ln CL$ of $m_{e,i} = 0$ from the background $m_e = 1e - 7$, $\Delta_{B,0}$. In windows where $\Delta_{B,0} > 0$, a model of strict divergence is more likely than an IM model. We highlight these barrier windows in red.
