# Supplementary material for: Genomic regions of current low hybridisation mark long-term barriers to gene flow in scarce swallowtail butterflies
Source: PLoS Genet. 2025 Apr 10;21(4):e1011655. doi: 10.1371/journal.pgen.1011655 (PMC12040345; doi:10.1371/journal.pgen.1011655)
Supplement: S7 Fig — (PDF) [file pgen.1011655.s009.pdf]

Dear Dr. Ebdon,
Thank you for submitting your manuscript to PLoS Genetics. It has been carefully reviewed by three referees. Overall, we appreciated your use of two innovative methods, gIMble and diem - one for demographic reconstruction and barrier locus mapping, and the other for chromosome painting in hybrids - within the same study. We also found your approach of comparing the results of these methods by analyzing parental populations far from the contact zone and hybrids within the contact zone interesting. In addition, we were intrigued by the result highlighted in your title, provided it was well supported. However, the reviewers have identified several concerns that need to be addressed before your manuscript can be considered for publication. Chief among these is the issue of small sample size, which was discussed by all of them. While the reviewers are open to the possibility that relevant conclusions can be drawn from a small sample, they remain unconvinced. About gIMble, while small sample size may not be a significant issue for inferring demographic parameters, it poses a challenge for barrier mapping. This aspect needs to be further explored and explicitly acknowledged in your manuscript. If your primary objective is to correlate the results with those obtained using diem, the effect of sample size may be less critical. However, it must be explicitly recognized that the positions of each individual barriers should not be considered strongly supported.

See our comment above and to reviewer 3 RE sample size. We have also added text to highlight that we have no strong support/resolution for the position of any particular barrier locus (which we believe is a general property of many sister species pairs).

Regarding the diem method, there is also a lack of clarity about the time scale to which your results apply.

This is a good point: one of the goals in fitting the tract length distribution to the model was to quantify the timescale. Our minimum estimate for the age of the Iphiclides contact is 150 - 275 generations (l. 367).

Given that your hybrid zone is likely partially congealed, and that only one individual could be considered an early generation hybrid, while the others are likely introgressed local parents, the congruence with the gIMble results may not be surprising. These local parents could simply be more introgressed, with bigger introgression tracts, compared to allopatric parents. The diem analysis again raises the question of whether your sample size is sufficient for conclusive results.

Our analysis (Fig 3) does indeed show that the HZ samples are more (and more recently) introgressed than allopatric samples and have bigger introgression tracts. It is not clear to us why/how this should affect the congruence with the gIMble result (which are based on different samples and estimates of gene flow over millions (rather than hundreds) of generations. Is the "*only one* individual" suggestion that a sample of *many* early generation hybrids would be more appropriate? It is difficult to see how that would be relevant, as early generation hybrids must be exceedingly rare in nature due to the width of the region around the HZ populated by complex hybrid-backcrosses. The "one individual" is not an early generation hybrid - it is a cross between two such complex hybrids.

In addition, you should acknowledge that your approach is conceptually similar, though not identical, to the genomic cline approach and discuss the relevant literature.

We have added text to highlight the difference between our approach (in terms of sample sizes and summaries) and cline fitting methods (l. 81-111, 299-305, 533-535). One important difference is that our method uses genomic information, i.e. the lengths of genomic ancestry tracts and a summary of admixture LD, while the “genomic cline” approach does not: it is simply the application of marker based population genetics to genome scale data. In fact, it may be argued that blockwise analyses represent the future direction of the genomic cline studies.

It is important to explicitly address these issues while explaining that your primary goal is the correlation shown in Figure 6. You must also propose a solution to ensure that local errors do not unduly influence the overall correlation.

Finally, as referee #2 pointed out, if the main objective of your study is the result shown in Figure 6, it needs to be discussed thoroughly. Was it expected or unexpected? How does it differ from the recent meta-analysis by Frayer and Payseur (https://doi.org/10.1093/evolut/qpae044) that found no correlation? It is important to discuss how your natural hybrids differ from lab-crossed hybrids in this context.

See 4) above

A small additional point. Figure 5 is not a De Finetti triangle and the concept of a genomic Wahlund effect is misleading. In an admixed population different loci will quickly drift or sweep to different allele frequencies and deviation from the line is more of an expectation than an exception. Please delete the dotted line. The difference between chromosomes is interesting, though, and underexplored. Some chromosomes support lower heterozygosity, what is their length? their density in barrier loci? what is their contribution to the result in figure 6?

Thanks for this suggestion. We have renamed the triangle plots ‘ternary plots of HI against H’ and removed the reference to a genomic Wahlund effect. We have added additional analyses to explore the relationship between D and chromosome length. We find a strong positive correlation between D estimated within windows and chromosome length (Fig S4C). This suggests that we see less gene flow across the hybrid zone on longer chromosomes which are more dense in barrier regions (l. 254) and less heterozygous (l. 248-250). We have retained the dashed line showing Hardy Weinberg equilibrium. We believe it is a useful reference point: for a sample of diploid genotypes taken from an individual in a spatial contact zone (as ours), HWE genotype proportions are the expectation if there is (1) per locality, per locus HWE and (2) all regions of the genome admix similarly. The latter is the null model* of both Barton's concordance and genome clines: our dashed line corresponds to this (double) null. Thus the deficit of heterozygosity in most HZ samples suggests local substructure/inbreeding in the HZ and/or selection against heterozygous (by source) ancestry which may be driven by partially dominant incompatibilities.

I'll stop here; you have a lot of comments to address in the reviewers' reports. It is imperative that you address all concerns in a thoroughly revised version of your manuscript. While there is no formal "reject/encourage" decision at PLoS Genetics, I am assigning your manuscript a major revision. Please be aware that while your analyses are of interest, PLoS Genetics places a strong emphasis on the novelty of a study, and I am not yet sufficiently convinced in

this regard after this round of reviewing. Therefore, it is crucial that your revised text meets the expectations of your title. Using gIMble and diem will not suffice.

You will also need to convince the reviewers that your methods are unbiased and that your sample size is adequate - neither of which is currently guaranteed.
I look forward to reviewing your revised manuscript.
Best regards,
Nicolas

Reviewer's Responses to Questions

Reviewer #1: In this study, Ebdon et al. describe barriers to gene flow between two species of Iphiclides butterflies at two different timescales, including the first genetic description of a hybrid zone between these species. They find a substantial barrier to gene flow that appears similar at both timescales. It is rare and difficult to compare these two timescales, and it was not a foregone conclusion that these approaches would identify the same loci within the genome. This manuscript utilizes two exciting, recently-developed techniques to draw inferences from an impressively limited number of individuals. This study will be exciting to many within the speciation community who are limited by sample collection. Additionally, the comparison of the distribution of tract lengths to theoretical predictions is particularly exciting.

Thank you for your enthusiasm for the novelty and interest of our study! We agree that one great benefit of our analyses of recent introgression over classic cline analyses is that it allows us to extract information about barriers from a small number of hybrid samples. We have added text to highlight this (l. 299-305).

A limitation of this work is the lack of a detailed recombination map for these species. The authors have addressed this in several ways, but it would be helpful to have two further questions clarified within the text: 1) Are the predicted relationships between chromosome structure and recombination rate on lines 202-205 supported by any literature within this genus?; 2) How do these results relate to the expectation that tracts should be broken down more slowly in regions of lower recombination, and thus we may see minor parent ancestry reduced at loci farther away from a barrier locus than in a region of high recombination? While the authors have already spent significant space on addressing the issue of recombination, I think it is important as it is key to some of the interpretations and would be relevant for other researchers who are unable to obtain a detailed recombination map for their species.

1) The chromosomal predictions are not supported by literature within the genus, given that *Iphiclides* only comprises our two focal species and a poorly known third species in Nepal. However, the *a priori* expectation that chromosome length is inversely related to recombination rate is supported by several direct estimates of butterfly recombination maps (e.g. in *Leptidea, Heliconius, Vanessa cardui*) as well many studies across a wide range of butterflies that have found chromosome length to be strongly correlated with genetic diversity which is indirect but strong evidence for the inverse relationship between chromosome length and recombination rate (Höök *et al*., 2023; Martin *et al*., 2019; Shipilina

*et a*l., 2022). We have clarified the correlation between genetic diversity and chromosome length in *Iphiclides* (l. 248-250)

2) We agree that we would expect the break-down of introgression tracts to be slower in low recombination regions. We stress that we do not claim to be able to separate the effects of selection against admixed ancestry and the recombination on short term barriers and have made this clearer in the text (l. 343-357). In particular, our analysis of the block length estimates a coupling coefficient theta = s/rho under the assumption that rho is uniform on the map, and so accounts for differences in r_{bp} at the level of chromosomes. In response to this comment we have added additional analyses to contrast the number of unique junctions (between ancestry tracts in HZ individuals) in long-term barrier windows with windows sampled at random (Fig 5E&F). This can be understood as a measure of the number of realised recombination events in the recent past.

Furthermore, it would be useful for the authors to discuss how the amount of divergence between the species reflects on the power to detect barriers along the genome, and if that could contribute to the observed similarities between the methods.

We thank the reviewer for raising this interesting point. We have added text to the discussion to explain that the time of divergence does not really matter for the power to infer barriers along the genome (l. 406-417). E.g. in *Heliconius melpomene/cydno* there is very little info about the divergence time and gIMble analyses under a model of equilibrium gene flow yield very congruent m_e profiles. In contrast, the power to detect barriers largely depends on the background level of m_e: lower overall m_e increases the FPR for deltaB0 barriers.

While this manuscript is well written, there are a few terms that require clarification when first introduced. In most cases, these terms are defined in the Methods (presented here following the Results), but not defined when first introduced in the Results. Examples include: “delta B0” on line 191, “focal barrier” on line 215, and the coupling coefficient terms on line 243. I recommend that the authors revisit the Results section and bring all necessary information forward from the Methods section, if it is to remain at the end of the manuscript.

We thank the reviewer for this comment. We have now provided extra clarifications and definitions for technical terms when they are introduced in the manuscript. In particular delta B0, focal barrier, and the coupling coefficient which we define in the same way as other studies (l. 343-347). We have rewritten the Results to provide a clearer explanation of the rationale for our choice of barrier metrics and have added two new metrics to measure the strength of short term barriers. We have also included two additional metrics of short term barrier strength: i) the density of high DI sites and ii) the number of unique junctions. We find that both yield qualitatively analogous results to D, i.e. we can show a significant overlap between short and long term barriers using all three metrics. The ring diagram of HZ barriers now includes a heatmap showing the density of high diagnostic sites (Fig 3).

Minor comments:

The authors should clarify the proportion of the genome that is reproductively isolated. The abstract says 33% and at line 193 it says 35%. If these numbers are not the same for a reason, it would be helpful to clarify.

Thanks for spotting this! The main text has been amended to 33%.

On lines 134 and 135, should these be references to Figure 1 rather than Figure 2?

These references have been amended to Figure 1.

Figure 5 is referenced in the text before Figure 4.

These have been switched.

Appendix equation A-3 appears to be missing a negative sign in the exponent.

Thanks for spotting this; this has been amended.

Reviewer #2: This paper compares to analyses of barriers to gene flow between two Iphiclides species. The first major analysis uses a nice recent IM approach to quantify effective rates of migration across the genome. The second method uses genomes from 6 (or sometimes 3) hybrids to investigate barriers that act currently. A central conclusion is the partial concordance between long- and short-term barriers.

Overall, I found this an interesting but frustrating paper to review. On the positive side, the new methods (Figs. 4-5) are exciting, and results from the IM methods are also interesting and well applied. On the negative side, the whole paper felt hastily written, with many methods inadequately reported (many Figures lack important information in the legends for example). I also thought that the paper lacked a clear message. As such, it was difficult to tell which results were meant as interesting descriptive asides, and which were meant to contribute to a broader conclusion.

1. Overall message

The final sentences of both the abstract and the conclusions seemed disappointing at the moment. The "aims and objectives" section currently names software packages and the four questions are just cryptic descriptions of the analyses that follow. The reader needs to understand exactly why it is important to compare long-term and short-term barriers. What exactly could we conclude if the two sets of barriers were congruent vs. incongruent? What would an intermediate degree of congruence tell us? None of this was made clear either from the introduction or the aims.

The reviewer states the "central conclusion is the partial concordance between long- and short-term barriers". Reviewer 3 also identified the same clear message: "to ask an interesting question in speciation genomics - the relationship between short term and longer term permeability of the genome to introgression". While we have certainly highlighted this conclusion (hence the title of our paper!), the previous version of the MS did not provide sufficient background to make it clear why this is an important question. In light of this comment, we have rewritten the introduction to set up this central question much more

clearly. In particular, we now discuss the recent attempt by Payseur et al (2024) to test for overlap between HZ barriers and incompatibilities identified in lab crosses (l. 81-94) and describe the processes that would lead to incongruence between short and long term barriers, e.g. reinforcement. We maintain that finding significant overlap in barriers acting across these very different timescale is an exciting and important result. We now also discuss avenues for deriving quantitative expectations for the overlap (l. 454-465).

2. Assumptions of the methods

The paper assumes that the reader is quite familiar with several previous papers (especially Barton and Gale 1993; Baird 1995; Laetsch et al. 2023, including e.g., the methods of parametric bootstrap). Currently, key quantities (S, R, D etc.) are not properly defined. I think more details of the methods should be reported throughout.

We thank the reviewer for this comment. We have revised the Results and Methods to provide additional information and clearer definition of technical terms as they are introduced in the manuscript. In particular, we have added information about both the parametric bootstrap implemented in gIMble (l. 587-608) and our new circular resampling scheme (l. 609-621).

3. Congruence between the two sets of barriers

An interesting claim of the paper is the congruence between the long- and short- barriers. This is demonstrated by a weakish negative correlation between m_e and D (reported just as a summary statistic), and by an ANOVA on D between barrier and non-barrier windows, (Figure 6).

If I am correct that this is the central result of the paper, the reader needs to be better convinced of its importance and robustness. The p value of the ANOVA is reported, but without summary stats, sample size, or tests of its parametric assumptions. We also need to understand exactly how independent the short- and long-term analyses are. Naively, we might expect analysis of recent hybrids to be confounded if the taxa had already undergone extensive gene flow in the recent past. Is this a problem or not.

We thank the reviewer for this comment which has prompted us to think more clearly about our statistical framework for testing the overlap between short and long term barriers. We believe that it is helpful to compare the full distribution in D for long-term barriers (hence Fig 6). However, we stress that an ANOVA or some form of standard linear model for comparing the properties of long term barriers and non barrier regions is inappropriate and would lead to vastly overconfident conclusions. The fundamental problem is that the basic unit of our analyses are genomic windows which are not statistically independent for two reasons: 1) overlap (we shift windows) and 2) autocorrelation due to LD and genomic properties. Other researchers have addressed these issues with a circular data resampling procedure (first described in Yassin et al. 2016 (https://www.pnas.org/doi/full/10.1073/pnas.1522559113). We now add a modification to this approach to sample random runs of windows from the data that have the same distribution between and within chromosomes as the observed outlier windows (see reviewer 3 below). Following this comment, we have used this modified

circular bootstrap to test for significance for all our analyses of barrier overlap and barrier correlates (e.g. Fig 5).

Another interpretation of the comment/question of robustness is whether our result of long term barrier overlap is robust to the choice of metric for short term barriers. Cline parameters are the obvious choice but not an option given the sample sizes available for this HZ system. We believe that D is an obvious/attractive measure. However, following this comment we realised that other measures are possible: 1) the density of diagnostic sites and 2) the number of unique junctions between long/recent ancestry tracts. Our main result of barrier overlap across timescales holds regardless of how we measure short-term barriers. We have added results for these alternative measures.

The conclusion also mentions possible confounding with recombination rate. I understand that a proper test might be impossible, but couldn't you, for example, compare results for short- vs long chromosomes?

Thanks for the suggestion. To check for the chromosome length effect we performed our circular bootstrap (see Fig 5 and response above) both across chromosomes and accounting for per chromosome resampling. This additional analysis shows that recombination rate variation between chromosomes contributes to but does not drive the observed overlap between short and long-term barriers.

Small points:

1. "we label windows as barriers to gene flow if a DIV history (me = 0) has greater marginal support than an IM history assuming the best fitting genome-wide value of me " Is this correctly described as "the strictest possible threshold" (p. 18), and might results be affected by adaptive introgression?

This is correctly described as the strictest possible threshold given that m_e =0 is a lower bound. We have now echoed the methods in this section of the results to clarify this difference in marginal support must also be supported by a parametric bootstrap. We would expect local m_e to be greater than the best fitting genome-wide m_e for windows affected by adaptive introgression so our definition for barriers is unaffected by adaptive introgression.

2."Genomic Wahlund effect" Are you suggesting an explanation here (e.g. that the reduced heterozygosity follows from spatial structure)? If so I would explain, if not, I would avoid the label.

Thanks for highlighting this. We now see that the interspecific heterozygosity deficit could be generated both by spatial structure and selection against admixture. We have modified the text to be clearer on this point (l. 286-295) and avoid the term Wahlund effect.

3. "The deficit of coding sequence is likely a consequence of the strong negative correlation between gene density and chromosome length (Pearson's ρ = -0.421) on one hand and the strong positive correlation between barrier density and chromosome length on the other. These together may explain the strong negative correlation between gene and barrier density (Pearson's ρ = -0.652), which is partially driven by the numerous small

chromosomes with very high numbers of genes and small numbers of barriers.”
Couldn’t this be tested?

Many thanks for this comment. We have now tested whether differences in recombination rate between chromosomes can explain the reduced cds fraction for barriers. Using a circular bootstrap scheme, we find that the deficit of cds for barrier windows cannot be explained by chromosome differences (Fig S3). Given that barriers are concentrated in the centre of chromosomes (which have a lower cds density) the possibility remains that the lower cds density of barrier windows is a by-product of a positive correlation between cds density and recombination rate within chromosomes.

Reviewer #3: Review of "Genomic regions of current low hybridisation mark long-term barriers to gene flow in scarce swallowtail butterflies"

This manuscript takes an interesting approach to make a lot out of a relatively modest data set to ask an interesting question in speciation genomics - the relationship between short term and longer term permeability of the genome to introgression. In contrast to the more traditional approach of comparing divergent population pairs at different points on "the speciation continuum" this study compares populations of the focal species pairs at different portions of the range - such that the allopathic populations reveal long-term historical introgression, while sympatric populations reveal the short-term recent process of hybridization and introgression. This is an interesting approach, which will undoubtedly inspire others to perform similar analyses in their systems. That said, I have numerous concerns about this study.

First - before asking their major motivating question, the authors make a solid approach to asking basic question about the system and inferring the history of speciation and gene flow. Notably they fit an IM model model to their allopatric samples and infer a history of ongoing gene flow. They also show that this history fits better than a simple model of pure divergence and that simple simulations under the best fitting demographic model of pure diverge do not confuse the IM inference. While this is reasonable, there are a few weaknesses here that must be noted, if not fully fixed.

First the details of simulation are quite vague - e.g. the authors say the used gable simulate to run these simulations. However this is not particularly informative as looking back at the gIMble paper, it seems that gIMble simulate is simply wrapper for msprime. As such the authors should a. Reference msprime as this seems to rob msprime of a citation and b. Provide more details about genome architecture - did they model the genome structure of their focal species? Or was this a bunch of unlinked sites? Or was this a few chromosomes all 1 Morgan long? Was the mutation rate fixed or variable across chromosomes etc etc. Similarly, it appears that the DIV model assumes a clean and instantaneous spell rather than split from a structured ancestral population etc etc etc. All of these details have important influence on the interpretation of the fact that neutral demographic simulations with a pure split of divergence are not confused for IM model as a misspecified demographic model can lead to poor inference. Similarly linked selection can also generate signals confused for introgression, especially in the IM framework [e.g. Smith and Hahn 2024https://academic.oup.com/genetics/advance-article/doi/10.1093/genetics/iyae089/7683793]. I'm not sure what to do with these criticisms, as the authors are using currently

established standard best practices of the field, but I am concerned that such issues could lead to incorrect inferences downstream.

Thanks for your comments. We have amended our citation to include msprime and have clarified this in the text (l. 589, 601). We have expanded our explanation of the simulation approaches in the methods (l. 587-608) and describe it below.

We performed the bootstrap simulation of the global model as follows: we used the parameters of the model of strict divergence which best fit the empirical data to parameterise the simulated demographic history. We simulated the observed number of 64 base blocks genome wide. We simulated 100 replicate datasets, each consisting of 6 diploid individuals per population to match the empirical sample set. We simulated a discrete genome and an infinite allele mutation model. We simulated a constant per base crossover rate assuming one crossover per male meiosis per chromosome. We assume a mutation rate of 2.9 e-9 as estimated in *Heliconius* (Keightley et al 2019). The grid-based simulations were done similarly, except that each window was parameterised with the best composite log-likelihood parameter set inferred from the empirical grid search and the window block length. We fit a strict divergence model and an isolation with migration model to each simulation and compared the distribution of improvements in fit to the empirical improvement in fit.

I have similar concerns about the interpretation of the block length distribution. The authors note some (but not all) of the complicating assumptions involved in going from a block length distribution (which they note is further enhanced by the lack of a genetic map) to an inference of selection. I don't think this analysis provides much support or anything, and it is probably best to remove it.

While our analysis of the block length distribution (necessarily) relies on simplifying assumptions, we do not share the reviewer's assessment that “this analysis does not provide much support of anything”. In contrast, reviewer 1 states “the comparison of the distribution of tract lengths to theoretical predictions is particularly exciting”. We would agree: we found the fact that the block length distribution can be fitted so well to this model DESPITE the simplifying assumptions involved remarkable. Contrary to the point of the reviewer, the most unrealistic/problematic assumption is not the uniformity in recombination rate within chromosomes but rather the underlying model which is extremely simplistic (hybrids backcross into a infinitely large population). Our study is the first attempt to apply this classic theory of blocklength distribution to HZ data. Our results highlight that this theory can indeed be used to learn about the relative magnitude of recombination and selection in empirical systems which we believe is an important finding in itself (we have added text to explain this better l. xx). Another important result is that our estimates of the coupling coefficient differ for the two directions of introgression and that this asymmetry agrees with the asymmetry in long term gene flow estimated for this species pair.

The most exciting result is - of course - the inferred correlation between long term and short term barriers to introgression.

This is an interesting result, but it was not well explained or motivated. Here are a few issues which require some though / attention:

We have rewritten the introduction to motivate this important result better. Please see our responses to reviewer 2 above.

First off the justification for using D - an indirect measure of admixture proportion rather than some more radiation approach was not well explained. Trying to read the minds of the authors, my intuition is that this reflects "mixture LD" (sense Falush et al 2003), and that the authors were worried that some more straightforward local ancestry deconvolution approach would miss admixed blocks on this short time scale. But this was neither stated nor supported / justified so it would help if the authors elaborated some.

We agree that our choice of D needed to be justified better and we have added text to make this clearer. We have also added two additional measures of short term barriers including the number of unique ancestry junctions (see our responses to reviewer 2 who also raised this point).

We are sceptical of the assertion that a local ancestry deconvolution approach would be more straightforward and maintain that D which is numerically equivalent to the variance in hybrid index across samples for a given length of sequence is both a simple and obvious summary of the strength of short term barriers that has intuitive limits: at the maximum (D=0.25), all samples are genotypically of one parental population or the other, at the minimum (D=0), all samples have a hybrid index of 0.5. Thus it provides a simple description of the degree of admixture (tending towards HI = 0.5, or towards HI = 0/1) given a set of samples and diagnostic markers. We are excited by the fact that using the number of unique ancestry junctions as a measure of short term barrier strength yields qualitatively analogous results (see new Fig 5E&F).

Second - the stats are not well explained - e.g. What is the ANOVA model for comparing barrier and non-barrier loci- is this simply a t-test or is there more to the model? What is done about the non-independence of windows ? My hunch is that both D and m_{e,i} are autocorrelated across the genome, so this should be addressed. Additionally, the stats reporting was strange - in one case a correlation coefficient was provided without a measure of significance, in another case a p value was provided without a summary of the effect size etc…

We thank the reviewer for this comment and agree that our stats on the differences between barrier and non-barrier windows were not well explained and inconsistent with our test for barrier correlates. We now use a circular data bootstrap test for these analyses (see our responses to reviewer 2 above) which we have implemented as a python script (l. 621, available at https://github.com/LohseLab/circular_bootstrap/).

Third - the biological question was unclear. Is this a question about the repeatability of key barrier loci, or a question about if the landscape of recombination itself is sufficient to generate correlations in local ancestry across time? Either way, the authors should better articulate their biological question, and either develop a parametric model that include variables such as chromosomes length, and position - -or develop some form of nonparametric matched permutation) to see if the correlation exceeds predictions off these simple correlates (although it could be that this model being inadequate simply reflects the

limited info about the genome etc). This is particularly important because the authors find that simple genome features well predict local introgression.

We have provided an alternative test for the difference between the distributions of D between barrier regions. We now resample random 'barrier' datasets of equivalent size to our empirical dataset 1,000 times in three ways. Firstly, we sample from the entire genome. Secondly, we resample per-chromosome datasets to account for the effect of recombination rate variation between chromosomes on our barrier strength distribution. Finally, we perform the resampling scheme but circularise the resampling (as described in response to reviewer 2 above). Our observed estimate of D in barrier regions is greater than the distributions generated by all three resampling frameworks.

Finally, the sample size here is remarkably small - only five samples from sympatry - and two with relatively little admixture. While it could be argued that making much of little data is a strength of the question and approach, I would like to have some sense of power, sample size necessary etc. While one genome provide a nice collection of coalescent genealogies and sometimes can be quite revealing for pop gen, one recently admixed sample does not. A stronger paper would include both some form of jacknifing at the individual level and some complimentary simulation.

As the reviewer points out, the sample size that is relevant for the power of coalescent based inference is not the number of sampled genomes but rather the number of genealogies (and ancestors involved) in their ancestry. By extension, the HZ individuals we analyse while recent on the coalescent time scale are NOT recent (or simple) in terms of recombination: the six HZ individuals we focus on contain ~ 8000 unique junctions, each corresponding to a crossover event between Ipod and Ifei ancestry. Thus, perhaps counterintuitively, our analysis is an order of magnitude more powerful than a typical lab study of barriers based on hundreds of second generation hybrids. We believe that the information contained in small samples of HZ individuals is worth highlighting (given that neither lab crosses nor large sample HZ studies are possible for most systems) and have added text to better explain this (l. 296-311).
