# Supplementary material for: Genomic regions of current low hybridisation mark long-term barriers to gene flow in scarce swallowtail butterflies
Source: PLoS Genet. 2025 Apr 10;21(4):e1011655. doi: 10.1371/journal.pgen.1011655 (PMC12040345; doi:10.1371/journal.pgen.1011655)
Supplement: Attachment — Submitted filename: Ebdon2024_responses_auresp_2.pdf [file pgen.1011655.s013.pdf]

Dear Dr. Ebdon,

Thank you for submitting your manuscript to PLOS Genetics. After careful consideration, we feel that it has merit but does not fully meet PLOS Genetics's publication criteria as it currently stands. Therefore, we invite you to submit a revised version of the manuscript that addresses the points raised during the review process.

Please submit your revised manuscript within 30 days Jan 04 2025 11:59PM. If you will need more time than this to complete your revisions, please reply to this message or contact the journal office at plosgenetics@plos.org. When you're ready to submit your revision, log on to https://www.editorialmanager.com/pgenetics/ and select the 'Submissions Needing Revision' folder to locate your manuscript file.

Please include the following items when submitting your revised manuscript:

* A rebuttal letter that responds to each point raised by the editor and reviewer(s). You should upload this letter as a separate file labeled 'Response to Reviewers'. This file does not need to include responses to formatting updates and technical items listed in the 'Journal Requirements' section below.
* A marked-up copy of your manuscript that highlights changes made to the original version. You should upload this as a separate file labeled 'Revised Manuscript with Track Changes'.
* An unmarked version of your revised paper without tracked changes. You should upload this as a separate file labeled 'Manuscript'.

If you would like to make changes to your financial disclosure, competing interests statement, or data availability statement, please make these updates within the submission form at the time of resubmission. Guidelines for resubmitting your figure files are available below the reviewer comments at the end of this letter.

We look forward to receiving your revised manuscript.

Kind regards,

Nicolas Bierne
Academic Editor
PLOS Genetics

Kelly Dyer
Section Editor
PLOS Genetics

Aimée Dudley
Editor-in-Chief
PLOS Genetics

Anne Goriely
Editor-in-Chief
PLOS Genetics

Additional Editor Comments:

Dear Dr Ebdon,
Your revised manuscript was examined by two of the three referees of the first round. The third did not respond to my request and I did not wish to invite a new referee. Both referees gave a positive feedback about this revised version. I share their positive evaluation.

We are happy to hear that you share the very positive evaluations of the two reviewers!

However, referee 1's concern about divergence time is shared by me, and it remains an important caveat of your paper.

Referee 1 corrects themself below "I was less concerned with the time of divergence and more concerned with the uneven density of diagnostic markers along the genome." We are grateful to the reviewer for raising the question of how/if our central result of long term/short term overlap depends on the heterogeneity in high DI variants. We have now included analyses of data downsampled such that the high DI variants used are uniformly distributed across each chromosome, all at the same density. Our results remain significant (see new Figs S8 & S9).

We are not convinced that your deductions and interpretations allow you to be so categorical in your conclusions about the contrast between two very different evolutionary time scales.

We are not sure who the "we" referred to here is. Neither referees 1 nor 2 doubts the value or validity of comparing barriers across two different time scales. In fact, in the first round of reviews all referees agreed that the timescale comparison is the most interesting and core result of our analysis.

Your poor knowledge of the study system, the limited number of demographic histories considered (2 pop IM model, no space, no variation of connectivity through time etc...), and above all the small number of individuals in a hybrid zone whose spatial structure and level of local introgression (the tails of the clines) are little known, do not allow you to assess whether the coincidence of regions resistant to introgression in and far from the hybrid zone is a surprise or not.

We are not sure we follow the arguments about model complexity and sample size here; we agree that the IM model is (necessarily) a drastic simplification and have pointed out in the discussion that the real history of *Iphiclides* is most likely one of repeated contact and isolation (l. 521-523). However, we maintain that despite these simplifying assumptions, modelling heterogeneity in m_e under the IM model is a useful way of capturing heterogeneity in long term gene flow.

Your junctions approach is the best you can get from this very small sample, and I very much appreciate it, but that's not the issue, the issue is more about the generality of the interpretations. So yes, you find figures, 100-200 generations in the ZH and 2M generations between the parents, but what?

We are relieved that the AE has no objection to our estimates *per se* and we do not understand the objection he has to our interpretation of these estimates, which is simply that we are estimating barriers that act over two vastly different timescales (the last 2 million vs the last 200 generations). What other interpretation of these estimates is possible?

Does it allow you to believe that you are doing better than previous studies that have attempted the same investigation? I receive, and share, and so do referee 1, the criticisms of lab crosses studies, but at least we know what type of hybrids we're studying. And I'm not very happy that you only make negative comments about the low number of recombination generations, as if all this literature should be thrown away, rather than trying to have a constructive discussion.

Having conducted studies of lab-generated F2 ourselves, we absolutely understand the value of controlled lab crosses. We are not suggesting at all that this literature should be thrown away and are sorry if our initial description of this important literature came across as overly negative (we have reworded and expanded this l. 102-114). However, the fact remains that i) lab crosses are simply not possible for many (if not most) young species pairs and ii) are limited in their power to detect/map barrier loci by the # of meioses/generations. Our approach of carefully studying variation in introgression across a natural HZ provides an alternative way to contrast the action of barriers across timescales.

In fact, I don't know whether I prefer 1,000 lab F2s or 6 hybrids from a little-known HZ, be they studied with an elegant junction method.

I could also well prefer a genomic cline analysis with less markers but many individuals and a nice transect.

We would argue that the question of what sampling design is preferable is moot, given that for many species that hybridise in nature, generating 1,000 F2 in the lab or sampling nice transects is not feasible anyway. We would argue that to obtain a general understanding of species barriers we have to make an effort to extract the maximum information from all systems.

The issue of possible post genome cline analyses is of course beyond the scope of this study. However, some fundamental problems with genome clines are described in this preprint: DOI: 10.22541/au.173953853.38181495/v1

In your case, you have your own vision of your complex HZ hybrids, but in reality we don't know very much (the fact you misunderstood my concern that you might have locally introgressed parents and an early generation hybrid between these introgressed parents, proves you have a preconceived view).

We are not sure what is meant here. Our inference of complex hybrids is based solely on the chromosome painting and not on any preconceived view/vision.

I don't share your certainties.

Again, we are unsure what certainties are being referred to here.

That said, contrary to my conclusion in the first round, I think your methodological approaches deserve to be published in PloS Genet, and the comparison of the two approaches remains super interesting.

We agree and would argue that this comparison is interesting precisely because it allows us to quantify the extent to which barriers acting over short and long timescales coincide.

However, I must ask you to make an effort to correct your certainties throughout your manuscript. You should work on the abstract and completely rewrite the author's summary. Throughout the manuscript, you must try to mitigate this very strong idea that you are comparing things that are as different as you say they are. If you have a secondary contact, even though the HZ introgression queues hybrids have recently incorporated new neutral heterospecific tracts, they are expected to have the same barriers as the parents. That's what people have done by comparing divergence islands and genomic clines in other studies. Your methods may well be more elegant, and that's where the novelty lies, not so much in the conceptual findings. I understand that my comment is somewhat at odds with my recommendation of the first round, but your revision and the very nice new analyses you've done, as well as your inability to really convince me of the significance of finding coincident barriers with the two methods, prompt me to ask you to tone that result down a bit in your revised version.

We have made the adjustments as laid out in our detailed responses to specific issues raised.

Sorry for the back and forth.I look forward to reading your revised version soon.

The first round of reviews has been really useful. Similarly, the current set of reviews has prompted an important sanity check: i.e. our finding of overlap between long and short term barriers is unaffected by heterogeneity in the density of high DI variants.

Best regards,
Nicolas Bierne

Minor comments :
- "vastly different timescales", "very recent signatures" "long-term signatures over the history of divergence" etc. please mitigate.

We are not sure what there is to mitigate. Our estimate of the onset of species divergence is ~ 2x 10^6 generations. Conversely, our lower bound for the age of the HZ is ~ 200 generations. Even if we ignore the HZ age estimate entirely and instead accept that an upper bound for the

age of the current secondary contact between the species is the current interglacial (~ 2 x 10^4 generations); i.e. two orders of magnitude more recent than the onset of species divergence. We would argue that our phrasing of “vastly different timescales” and “recent vs long-term” barrier effects is justified. We have made the timescale difference clearer in the text and have replaced “very recent” with “recent”.

- L46 "speciation in the face of gene flow" → episodes of gene flow during speciation

Ok, we have changed this.

- L58 "contemporary contact zones are likely one of many instances of secondary contact generated by drastic environmental changes" : I don’t understand what you mean here, probably too much than what you could.

We simply mean that glacial cycles (and the current ranges of the two species) give us a strong prior for episodes of gene flow (see immediately previous comment), i.e. repeated cycles of secondary contact (during interglacials) and isolation (in refugia during glacial period), which is the same point the AE raised above. We have reworded this to make this clearer (l. 57-60).

- L 173 "An IM model supports a history of speciation with gene flow". What can an IM model support other than an IM model? Given you do not test secondary contact or varying level of geneflow with time, do not conclude more than what you can. I realy don’t mind that you use an IM model to map the barriers, but do not conclude too much about the history of divergence and gene flow. That a hybrid zone in the southwest of France would not be a secondary contact after post-glacial recolonization would surprise me a lot.

We absolutely agree that the *Iphiclides* HZ is most likely the outcome of secondary contact (see the comment above). We have changed the subsection title and rephrased this to make it clearer that we do not claim that our support for the IM allows us to say anything about the relative fit of continuous (vs episodic) gene flow over time. However, we are interested in quantifying the heterogeneity of average (since the onset of species divergence) gene flow along the genome and the IM model is the simplest scenario that captures long term average m_e. See also our comments at the top.

- Figure 4 "The dashed line indicates the expectation under Hardy-Weinberg equilibrium" Again, please delete this dashed line. If I make an effort to go your way, this line could be useful if you plot the population average (but you don’t really have a population sample here), that this average is close to the line, and you find a way to show how far the individual scatterplot deviates from it as a measure of HW and linkage disequilibrium (but the variance in the ancetry does the job well). When you compare different parts of a genome, it's against the genome average, but given that an individual genomic average may well deviate from the line due to linkage disequilibrium in HZ or selection and drift in an admixed population, I don't see the point of showing that expectation. Finally, as I said it previously, in an isolated admixed population that converged toward HWLE, one might expect individuals to be close to this line under

neutrality and slow drift in large populations, but this is never the case, you always deviate from it (notwithstanding that theory predicts selection for heterozygosity and ancestry of the major or fitter parent). When an expectation is not useful, when it is not well explained and when it can mislead the reader because it is unusual, I don't see the point of showing this expectation. To put it briefly, I don't agree with you at all that it's a useful expectation.

The nature of the expectation is now explained in the main manuscript:

"Interestingly, all HZ samples except 1322 show a deficit in interspecific heterozygosity relative to Hardy Weinberg equilibrium (Fig \ref{fig:tritriangle}). While these ternary diagrams show genotype samples from genomes rather than from loci, Hardy Weinberg equilibrium remains the null expectation for localities at equilibrium for an admixture process uniform along the genome. Thus the deficit in heterozygosity suggests local substructure/inbreeding in the HZ and/or heterogeneous selection against heterozygous (by source) ancestry which may be driven by partially dominant incompatibilities." (l. 300-306)

It is straightforward to show this is the case (see the attached.cdf document which can be read using the free ‘player’ from Wolfram URL).

Journal Requirements:

1) Please amend your detailed Financial Disclosure statement. This is published with the article. It must therefore be completed in full sentences and contain the exact wording you wish to be published. Please ensure that the funders and grant numbers match between the Financial Disclosure field and the Funding Information tab in your submission form. Note that the funders must be provided in the same order in both places as well.

- State the initials, alongside each funding source, of each author to receive each grant. For example: "This work was supported by the National Institutes of Health (####### to AM; ###### to CJ) and the National Science Foundation (###### to AM)."

- State what role the funders took in the study. If the funders had no role in your study, please state: "The funders had no role in study design, data collection and analysis, decision to publish, or preparation of the manuscript.".

We have now stated this.

If you did not receive any funding for this study, please simply state: u201cThe authors received no specific funding for this work.u201d

Reviewers' comments:

Reviewer's Responses to Questions

Comments to the Authors:
Please note here if the review is uploaded as an attachment.

Reviewer #1: In this study, the authors investigate short- and long-term barriers to gene flow between two species of Iphiclides butterflies. They use two recently developed techniques (gIMble and diem) to draw inferences from small sample size. Interestingly, they find that the barriers identified through the two approaches overlap significantly. They find that the Z chromosome has very little introgression. This study is exciting because such comparisons of different times scales are rare.

We thank the reviewer and agree that finding concordance of barriers across timescales is our most exciting & novel result.

This is the second time I have reviewed this manuscript. I appreciate the authors' efforts to make improvements, and I believe the manuscript has been strengthened as a result. I appreciate the new emphasis on the overlap between the short- and long-term barriers, which is a novel contribution of this manuscript. I particularly appreciate the authors' addition of junction number as a metric and their new permutation approach, as it is exciting to see this theory applied to real data. Previous attempts at applying junctions to real data have looked at very different types of hybrid populations that made this comparison difficult (e.g. Lavretsky et al 2019, https://doi.org/10.1002/ece3.4981).

We thank the reviewer for these comments.

That being said, I'm not sure that the authors have addressed my concern about divergence. I was less concerned with the time of divergence and more concerned with the uneven pattern of differentiated markers along the genome. At line 330, the authors state that "sites with high diagnostic indices are more densely populated within barrier windows relative to a null resampled distribution." Is there an issue of relative power along the genome? It would improve the paper for the authors to address this.

To address this issue, we have now included analyses of data downsampled such that the differentiated markers used are uniformly distributed across each chromosome, all at the same density. Our results remain significant, showing that, while indeed there was originally more power in some regions of the genome, this is not what drives our results (see Figs. S8 & S9).

Minor comments:
1) I agree with the authors that the Frayer and Payseur paper has several additional complications brought on by the lab vs wild cross comparison (as was the purpose of their discussion), but it may also be worth noting that the time scales are different (where late-stage hybrids in that comparison may be closer to the short-term barriers considered here).

This is a good point; we have made it clearer that these are different timescale comparisons (l. 97-102): our short time-scale barrier measure is closer (in generations) to the long timescale of Frayer & Payseur. Whether this means closer (in effect on barrier evolution) is an interesting question.

2) Around line 205, it would improve clarity to explicitly say that the loci used for the sliding windows are not the same as those used for fitting the global IM model above.

We have made it clearer that sliding windows are constructed from blocks (l. 219).

3) At lines 332-334, you say that the number of junctions should be negatively correlated with barrier strength. While I agree a reduction is expected, I think there is evidence that the strength of the reduction is modulated by several factors and not just selection strength (e.g. Hvala et al 2018, https://doi.org/10.1111/evo.13509). If this is based on specific theory, it would be good to cite it. This does not impact your conclusion, as you only show a reduction and not a correlation.

Thank you for bringing the link to this publication to our attention, we have now referred to it in the text.

4) At lines 516-518, the authors state that their approach may undercount small blocks, but they actually observe more small blocks than expected by their model. Please clarify this point.

Thank you for catching this contradiction. Text changed:

"This has an advantage over HMM approaches in that it has no starting point chirality, but the disadvantage that it censors true small tract signal with some distribution of false negatives. This if anything makes the excess of small tract observations over simple expectations more surprising (Fig 6)." (l. 538-541)

5) On lines 583 and 596, the authors reference different Keightley papers for the same mutation rate.

Thanks for catching this. We've fixed the reference.

6) In the Methods section titled "Untangling blocks from genotypes," the authors state that no phasing choice is necessary to determine the lengths of minor tracks of introgression. While this is true, a choice would still be required in most cases where minor ancestry is homozygous. The legend of Figure 6 suggests that that does not happen in most cases, and this seems reasonable given the relative proportions of admixture. However, it would be useful to clarify in the Methods both that most blocks were heterozygous, and what was done with homozygous blocks.

A good point. We have modified the Methods to clarify.

"When diploid genomes are polarised with respect to a single barrier, an interval of heterozygous sites within otherwise homozygous chromosome genotype suggests a single tract

of minority inheritance on one of the chromosome copies. While the same pattern would result from two minority tracts, one on each chromosome copy, that 'perfectly' abut (without overlap), simple genotypes can be parsimoniously (and automatically) parsed into haplotype tracts by assuming such perfect abutment is so rare as to be negligible.This accounts for the vast majority of the data: for each such heterozygous a block on one strand is counted. Occasional homozygous introgressed intervals are treated similarly: a block on one strand is counted. Finally (and rarest of all), intervals of mixed homozygous-introgressed and heterozygous state are treated similarly: a block on one strand, spanning the mixed interval, is counted" In this way small blocks which positionally overlap with larger introgressing blocks within the same diploid individual are censored from the block size distribution." (l. 683-694)

7) At the end of the Figure 5 legend, there should be a reference to plot F rather than plot E.

Thank you, this has been amended.

Reviewer #2: This manuscript is greatly improved. I like the new stats and especially the new circular randomization. The intro and discussion also do a much better job of contextualizing the work, and explaining why its questions are interesting. I am grateful to the authors for their constructive response to the earlier review.

We are also grateful for the reviewers thoughtful and constructive comments in the first round of reviews

I have two very very small comments:

1. There are lots of acronyms and their component single letters in this ms (D, Z, I, H, M, HI, DI, HZ, IM, IF, HWE, LD, CL etc.). I know they are all standard, but I would double check if any of the rarer ones could be removed (e.g. mt, x, HWE, FPR), or different notation used when similar symbols appear in the same paragraph (e.g. p. 9 has H, I, Z, HZ, HI, and IF).

Thank you, we have decided to remove mt, HWE, and FPR given their infrequency. Given IF is part of a sample name code we hope that the co-occurrence of HI and HZ is not too confusing.

2. p. 6: "using a pre-computed grid" Not sure what this meant.

We have modified the text (->using a precomputed grid of likelihoods) to make this clearer

---

Have all data underlying the figures and results presented in the manuscript been provided?
Large-scale datasets should be made available via a public repository as described in the *PLOS Genetics* data availability policy [track.editorialmanager.com], and numerical data that underlies graphs or summary statistics should be provided in spreadsheet form as supporting information.

Reviewer #1: Yes

Reviewer #2: Yes
